# Comprehensive SNP array study of frequently used neuroblastoma cell lines; copy neutral loss of heterozygosity is common in the cell lines but uncommon in primary tumors

**DOI:** 10.1186/1471-2164-12-443

**Published:** 2011-09-07

**Authors:** Hanna Kryh, Helena Carén, Jennie Erichsen, Rose-Marie Sjöberg, Jonas Abrahamsson, Per Kogner, Tommy Martinsson

**Affiliations:** 1 Department of Clinical Genetics, The Sahlgrenska Academy, University of Gothenburg, Sahlgrenska University Hospital, SE-41345 Gothenburg, Sweden; 2 Department of Pediatrics, The Sahlgrenska Academy, University of Gothenburg, Sahlgrenska University Hospital, SE-41685 Gothenburg, Sweden; 3 Childhood Cancer Research Unit, Department of Woman and Child Health, Karolinska Institute, Karolinska Hospital, SE-17176 Stockholm, Sweden

## Abstract

**Background:**

Copy neutral loss of heterozygosity (CN-LOH) refers to a special case of LOH occurring without any resulting loss in copy number. These alterations is sometimes seen in tumors as a way to inactivate a tumor suppressor gene and have been found to be important in several types of cancer.

**Results:**

We have used high density single nucleotide polymorphism arrays in order to investigate the frequency and distribution of CN-LOH and other allelic imbalances in neuroblastoma (NB) tumors and cell lines. Our results show that the frequency of these near-CN-LOH events is significantly higher in the cell lines compared to the primary tumors and that the types of CN-LOH differ between the groups. We also show that the low-risk neuroblastomas that are generally considered to have a "triploid karyotype" often present with a complex numerical karyotype (no segmental changes) with 2-5 copies of each chromosome. Furthermore a comparison has been made between the three related cell lines SK-N-SH, SH-EP and SH-SY5Y with respect to overall genetic aberrations, and several aberrations unique to each of the cell lines has been found.

**Conclusions:**

We have shown that the NB tumors analyzed contain several interesting allelic imbalances that would either go unnoticed or be misinterpreted using other genome-wide techniques. These findings indicate that the genetics underlying NB might be even more complex than previously known and that SNP arrays are important analysis tools. We have also showed that these near-CN-LOH events are more frequently seen in NB cell lines compared to NB tumors and that a set of highly related cell lines have continued to evolve secondary to the subcloning event. Taken together our analysis highlights that cell lines in many cases differ substantially from the primary tumors they are thought to represent, and that caution should be taken when drawing conclusions from cell line-based studies.

## Background

Neuroblastoma (NB) is the most common extracranial solid tumor of childhood, a malignancy believed to arise from primitive cells of the sympathetic nervous system. This disease is characterized by a high degree of heterogeneity, ranging from spontaneously regressing growth to highly malignant tumors [1]. The most common genetic abnormalities found in aggressive NB tumors are partial deletion of chromosome arms 1 p and/or 11 q, gain of genetic material for chromosome arm 17 q and amplification of the proto-oncogene *MYCN *[1-5]. Several different types of array based methods have been used over the past few years for assessing copy number changes on a genome wide scale in NB tumors [4-14]. The resulting pattern of genetic alterations is used today to classify the tumors into prognostic subgroups, indicative for the treatment of the patients. Generally, tumors with a near-triploid appearance, containing only numerical aberrations (i.e gains and losses of whole chromosomes), have a very good prognosis, while tumors with MYCN amplification and tumors with 11 q deletion constitute two groups with unfavorable prognosis and poor survival. Both these latter groups typically also present with 17 q gain [4,5]. Furthermore, we have found that tumors presenting with 17 q gain, but without MYCN amplification or 11 q deletion, form a group with intermediate prognosis, and that tumors presenting with other segmental aberrations (i.e without MYCN amplification, 11 q deletion or 17 q gain) have a favorable prognosis [4]. In this study we have used high density single nucleotide polymorphism (SNP)-arrays from Affymetrix. These arrays have the advantage over BAC-based array-CGH that hybridization intensities are recorded also for each allele of an SNP, which gives simultaneous information on both LOH and copy number status. To ensure proper detection of LOH regions in the case of stromal cell contamination or clonal differences within the tumor, we have used the AsCNAR algorithm available in CNAG 3.0 [15]. When calculating the allele specific intensities, this algorithm takes advantage of the false heterozygous calls that might appear as a result of contaminating normal cells [16]. This makes it a robust method for visualizing LOH and other allelic imbalances even in complex tumor materials.

One type of alteration that typically goes unnoticed using conventional array-CGH platforms is copy neutral loss of heterozygosity (CN-LOH), a special case of LOH occurring without any resulting loss in copy number. This phenomenon might; (i) be a case of identical by descent (IBD) i.e. two identical copies of a particular region are inherited independently of each other due to parents that are closely related to each other, (ii) be an effect of uniparental disomy (UPD), i.e. both copies are derived from the same parent, as in the Prader Willi and Angelmans syndromes or (iii) be a tumor acquired event where a homozygous situation is achieved by losing one copy and then compensating for this with duplication of the other allele. This is most often denoted in the literature as CN-LOH, or sometimes also as acquired UPD. In this article we refer to CN-LOH as the tumor acquired form and specifically use IBD or UPD when appropriate.

CN-LOH is sometimes seen in tumors as a way to inactivate tumor suppressor genes, and has been reported in many different types of cancer [17]. Here we present data regarding the frequency and appearance of copy neutral events and other allelic imbalances in a panel of 134 primary NB tumors and 10 NB cell lines. We also present full genomic profiles for 10 NB cell lines and discuss evolutionary differences between related cell lines.

## Methods

### Samples and DNA preparation

134 primary NB tumors from Sweden, 66 of stage L, 51 of stage M, 4 of stage MS and 13 of unknown stage, were included in this study. The stages are according to the recently introduced INRG classification [18]. Ten NB cell lines were also analyzed; IMR32, Kelly, NB69, SH-SY5Y, SK-N-AS, SK-N-BE(2), SK-N-DZ, SK-N-FI, SK-N-SH (ECACC, HPA Culture Collections, Salisbury, UK) and SH-EP (ATCC, Manassas, VA), as well as constitutional DNA from 30 healthy blood donors. The primary tumors were histologically assessed for tumor cell content using adjacent tumor tissue to that used for DNA extraction, and total genomic DNA was then isolated using DNeasy blood and tissue kit (Qiagen, Hilden, Germany) according to the manufacturer's instructions. Informed consent was retrieved from the parents of the patients. Ethical permission was granted by the local ethics committee (Karolinska Institutet and Karolinska University Hospital, registration number 03-736 and 2009/1369).

### Cell line validation

STR typing of the cell lines was performed using the AmpFLSTR^® ^Identifiler^® ^kit (Applied biosystems; i.e. the so called CODIS kit) according to the user manual (PN 4323291) and the validation report (Applied Biosystems 3730 DNA Analyzer Human Identification Validation Report, October 2007) with the following changes: PCR amplification was run in 10 μl reactions in a 384well format, using 2ng DNA as template and 25 cycles of annealing/elongation. Heat sealing film was used to seal the plates and the amplification was run in a GeneAmp^® ^PCR System 9700 without 9600 emulation, followed by fragment analysis using a 3730 DNA analyzer with POP7 polymer and 10s injection time. GeneScan500 LIZ^® ^was used as size standard and AmpFLSTR^® ^Identifiler^® ^Allelic Ladder as well as a control DNA (Q9947A) was always run in parallel to the samples. GeneMapper v3.6 was used for analysis with peak window size set to 11 points. STR genotype profiles for each cell line were compared with that of the Children's Oncology Group (COG) Cell Culture and Xenograft Repository web page (http://strdb.cogcell.com/).

### High resolution SNP array analysis

The tumors where analyzed for copy number changes and LOH using either Genechip^® ^Human Mapping 50K XbaI (22 samples) or Genechip^® ^Human Mapping 250K NspI (112 samples; Affymetrix Inc., Santa Clara, CA). A number of these cases have been presented before [3,4], albeit analyzed for other features. The experimental procedure has been previously described [3].

### Data analysis and software

GeneChip^® ^operating software (GCOS) and GeneChip^® ^Genotyping Analysis Software (GTYPE;Affymetrix) were used for primary data analysis, normalization against internal control features on the chip, genotype calling and quality control. Subsequent analysis was then performed using Copy Number Analyzer for GeneChip (CNAG 3.0;Genome Laboratory, Tokyo University) [19] featuring the algorithm for Allele-specific Copy-Number analysis using Anonymous References (AsCNAR) [15,16]. In CNAG, the tumor samples were compared in silico to the 6 best matched control samples (lowest standard deviation) available among a set of non-matched healthy individuals. This set contained both HapMap samples available from Affymetrix as well as our own set of healthy control samples.

### Fluorescent In Situ Hybridization (FISH) analysis

FISH analysis for chromosome 1 (1pter and 1cen), 2 (*MYCN*) and sometimes 17 and/or 18, has been performed on most of the tumors in the NB material for diagnostic purposes. The experimental procedure has been described elsewhere [20]. The available FISH-data was used as a comparison for the copy number estimations from CNAG on a per chromosome basis.

### Multiplex ligation-dependent probe amplification (MLPA)

MLPA analysis for copy number changes in the CDKN2A/2B region at 9p21 was performed using the Salsa MLPA Kit P024B (MRC-Holland, Amsterdam, the Netherlands). The analysis was performed according to the protocol provided by the supplier with some minor changes [3].

### Statistical analysis

Fisher's exact test was used to compare the proportions of samples presenting with at least one near-CN-LOH event while the actual frequency of near-CN-LOH events was compared using the Mann Whitney U test. The near-CN-LOHs were also divided into three different subcategories; interstitial (small, segmental events within the chromosome), telomeric (segmental events extending to one end of the chromosome) and whole chromosome events (including the entire chromosome). For simplification the × chromosome was excluded, and the cell-lines SH-EP and SH-SY5Y were also omitted from the analysis due to their shared ancestry with the cell line SK-N-SH. Array profiles indicative of a high content of stroma cell contamination, i.e. cases without any visible aberrations (flat profile), with MYCN amplification only or with CN-LOH only, were also removed from the analysis.

## Results

### Copy number estimations in complex tumors

**Figure 1:**
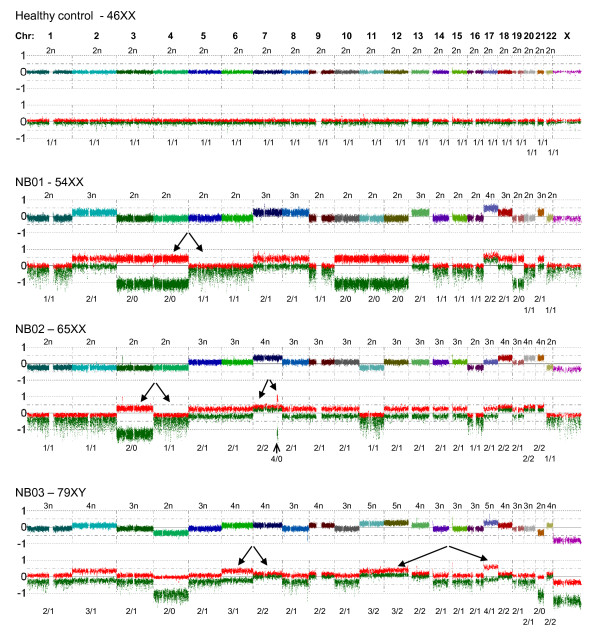
**Estimating ploidy number in NB tumors with complex genomic profiles**. Allele specific intensities, calculated using the AsCNAR algorithm, makes it possible to estimate the copy number for each chromosome in complex tumors. Arrows indicate differences seen only on the allele specific level that would have been overlooked or misjudged using other methods, for example 1+1 vs. 2+0 or 2+1 vs. 3+0. Several examples of near CN-LOH is also shown; chromosomes 3, 4, 10, 11, 12 and 19 all show an allelic distribution of 2+0 in NB01 which has an overall ploidy of ~2,3n, and chromosome 3 in NB02 has an allelic distribution of 2+0 in an otherwise near triploid tumor (overall ploidy ~2,8n). The multi-colored line shows the total copy number for the different chromosomes and the red and green lines show the strongest and weakest allele intensity respectively. Total copy number is written above each chromosome and allele specific copy numbers are written underneath the allele intensity plot as: red/green.

The NB tumors and cell lines were analyzed with respect to copy number change and LOH using CNAG 3.0. General ploidy information was also derived from these array data, and in many near triploid cases the copy number for the individual chromosomes differed widely. Although this posed some difficulties in assessing the overall ploidy, the allele specific intensities from the AsCNAR algorithm still made it possible to estimate the copy number on a per chromosome basis, even for the more complex triploid or tetraploid tumors (Figure 1). This was achieved by sequentially comparing the total copy number for each chromosome with the corresponding allele based intensities, thus trying to fit the most appropriate copy number to each intensity level. The estimated copy numbers were then compared to FISH data, whenever available. Although we only had access to FISH data for a few chromosomes, it correlated well with the copy number estimates from CNAG and only a few cases needed to be revised due to tetraploid tumors being erroneously classified as diploid tumors.

### What is copy neutral in a near triploid tumor?

When these polyploid tumors were analyzed regarding CN-LOH it was found that many different types of LOH exist in these tumors that are not exactly copy neutral, nor are they representing classical hemizygous deletions. Due to the AsCNAR algorithm, these regions of LOH are clearly distinguished from regions with a maintained heterozygosity, even in complex triploid or tetraploid tumors (Figure 1). These findings brought up the question whether CN-LOH is a relevant measure in these tumors, and led us to broaden the definition of CN-LOH to include all LOH that are at least partially compensated by the remaining allele, from now on referred to as "near CN-LOH". Thus, in a near-triploid tumor, allelic distributions of 2+0 or 3+0 are both considered as "near CN-LOH". Additionally, other allelic imbalances not involving LOH, such as allelic distributions of 3+1 vs. 2+2 or 4+1 vs. 3+2 were identified in the tumors, but since most of these cases are imbalances rather than true loss of heterozygosity, they were not included in the statistical calculations.

### Frequency of near CN-LOH in NB

35% (47/134) of the primary NB tumors presented with at least one region of near-CN-LOH, compared with 87% (7/8) of the NB cell lines (p = 0.005). The actual frequency of near-CN-LOH was also found to be higher in the cell lines (median: 2 events; mean: 2.4; 25th-75th percentiles: 1-3.5) as compared to the primary tumors (median = 0 events; mean: 0.89; 25th-75th percentiles: 0-1) (p = 0.002). Since the near-CN-LOH seen in the primary tumors might be due to IBD, a comparison was also made between the primary tumors and the set of healthy controls. The amount of near-CN-LOH was higher in the primary tumors (median: 0 events; mean: 0.89; 25th-75th percentiles: 0-1) as compared to the controls (median: 0 events; mean: 0.10; 25th-75th percentiles: 0-0) (p = 0.005), indicating that the frequency of CN-LOH in the tumors is not solely accounted for by IBD events.

The three subtypes of CN-LOH (interstitial, telomeric, whole chromosome) were also found to differ in abundance between the groups. Among the cell-lines the by far most frequent forms of CN-LOH were telomeric (74%), followed by whole chromosome events (21%) and interstitial events (5%). The healthy controls on the other hand only had the interstitial form of CN-LOH (100%), while the primary tumors were somewhere in between, with mostly interstitial events (50%) followed by whole chromosome events (35%) and telomeric events (14%).

Furthermore, a comparison was made between the amount of near-CN-LOH in the tumors and the outcome of the patients, defined as either dead of disease or no evidence of disease, i.e. alive and with an overall survival of at least 5 years. However the number of near-CN-LOH events was not found to differ between the two outcome categories, regardless of the CN-LOH subtype studied.

### Distribution and chromosomal locations of near CN-LOH in NB

**Figure 2:**
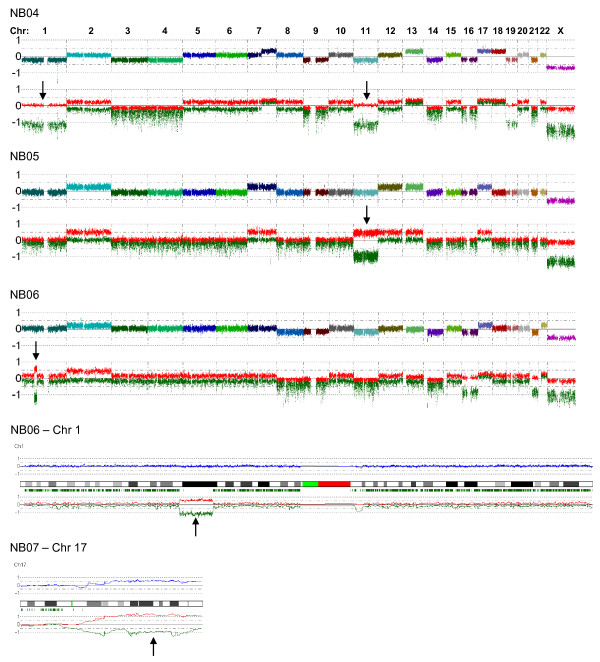
**Near-CN-LOH in clinically interesting regions**. Arrows indicate clinically interesting regions with near-CN-LOH (NB04, NB05 and NB06) or gain-LOH (NB07). The CN-LOH in NB06 is shown in both genome-wide view and single chromosome view for comparison. Total copy number is depicted as a multicolored or blue line in genome-wide view and single chromosome view respectively. The red and green lines show the strongest and weakest allele intensity respectively for both views.

**Figure 3:**
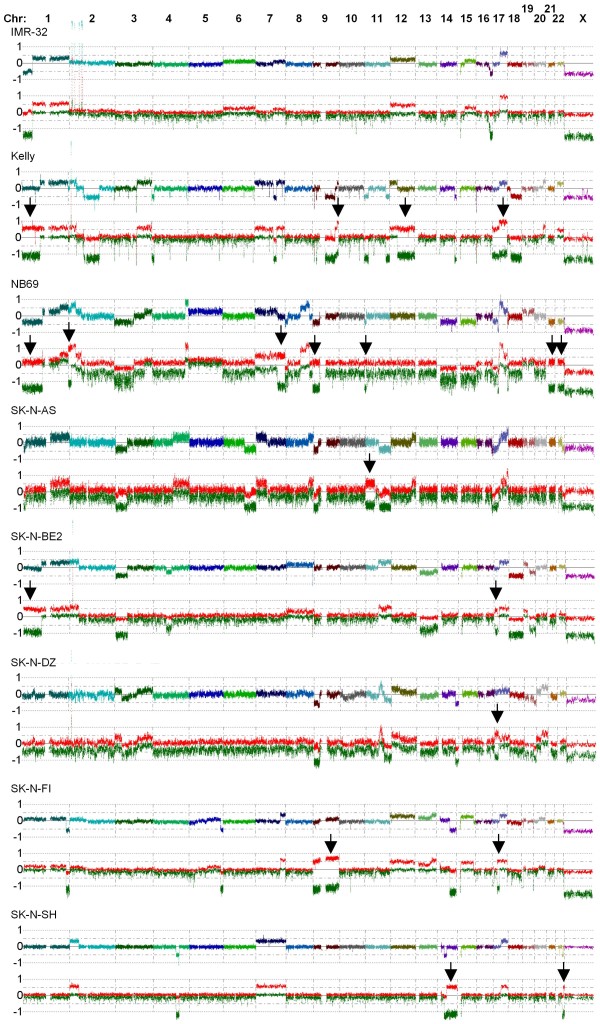
**Allelic imbalances in NB cell lines**. Genome-wide chromatograms are shown for all the NB cell lines analyzed. Arrows indicate regions of near-CN-LOH or Gain-LOH. The multicolored line shows the total copy number for the different chromosomes and the red and green lines show the strongest and weakest allele intensity respectively.

The distribution of near CN-LOH in the tumors seems to be relatively even across the genome, with all chromosomes affected in at least one tumor sample, except for chromosome 20 (data not shown). Some of the near-CN-LOHs in the primary tumors were found in the regions of 1 p and 11 q and some cases of gain-LOH were also found in the region of 17 q (Figure 2). These three regions are thought to be of clinical interest and are often affected with loss or gain in aggressive NB tumors. The cell lines also display a distribution of near-CN-LOH that is fairly scattered, although the overall frequency is increased compared to that in the primary tumors. Genome-wide array profiles are shown in Figure 3 for all cell lines examined.

### Genetic differences between closely related cell lines

**Figure 4:**
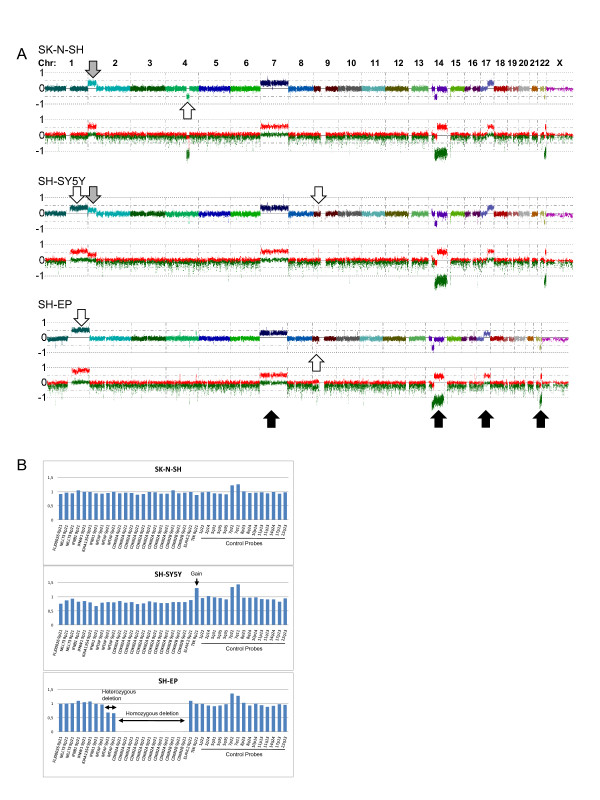
**Clonal differences between cell lines**. A) Genome-wide chromatograms for the highly related cell lines; SK-N-SH, SH-EP and SH-SY5Y. Aberrations shared by all three cell lines are shown as black arrows, those that are shared by two of the cell lines are shown as grey arrows and the ones unique to each cell line are shown as unfilled arrows. Note that all three cell lines have acquired new aberrations since the sub cloning took place. The gains seen at 1 q for SH-SY5Y and SH-EP, although similar in this picture, are not identical in position and copy number and probably represent two different events. Only aberrations larger than 1.5 Mb are indicated in the figure, with the exception of the homozygous deletion at 9p21 in SH-EP. B) MLPA results confirming the deletion at 9p21 found in SH-EP, as well as the small gain found in SH-SY5Y in this region. Neither of these aberrations can be seen in the parental cell line SK-N-SH. The whole chromosome gain at chromosome 7, seen in all three cell lines, is also confirmed by the MLPA analysis.

The cell line SK-N-SH has been described as consisting of two distinct morphological cell types, one neuroblast-like and one epithelial-like, and sub-clonal cell lines have therefore been established from SK-N-SH in several subsequent steps [21]. We compared the array profiles for two of the resulting cell lines; SH-EP (epithelial-like) and SH-SY5Y (neuroblast-like), to each other and to the array profile of the parental cell line SK-N-SH in order to identify possible genetic differences that have occurred during the sub-cloning (Figure 4).

The three cell lines were found to be strikingly similar and shared many of the aberrations, for example trisomy for chromosome 7 and partial CN-LOH at 14 q and 22 q. One aberration (2 p gain) was also found to be shared between the parental cell line and one of the daughter cell lines (SH-SY5Y). However, several unique alterations were also found for each of the cell lines (Figure 4 and Additional file 1), for example in the SH-EP cell line a homozygous deletion was detected at 9p21.3 in the region containing the genes *CDKN2A *and *CDKN2B*. This deletion, as well as a small gain found in a nearby region in SH-SY5Y was confirmed using MLPA (Figure 4b). Interestingly, although a gain at 1 q is seen in both daughter cell lines (SH-SY5Y and SH-EP), they do not share the same breakpoints, and the actual copy number also differs, with three and four copies respectively. Thus although the alterations look fairly similar, they seem to be the result of two different events. Finally, not only does the daughter cell lines contain unique aberrations, there are also a few alterations found to be specific for SK-N-SH itself, for example an interstitial deletion at 4 q which partly overlaps with another smaller interstitial deletion, common to all three cell lines, thus causing a homozygous deletion in the overlapping region.

All ten cell lines were also validated using STR genotyping, and the resulting profiles were compared against public profiles at the Children's Oncology Group database. All our cell lines were found to match with the previously reported profiles, and SK-N-SH, SH-EP and SH-SY5Y were additionally found to have identical STR-profiles thus confirming their shared ancestry (data available on request).

## Discussion

### Frequency of CN-LOH in NB

CN-LOH has previously been reported in many types of cancers [17] and to some extent in neuroblastoma [3]. However the amount of LOH that is truly copy neutral found among primary NB tumors is rather low compared to many other tumors [17]. In this paper we report a frequency of near-CN-LOH in primary tumors (35%) that differs from our previously reported frequency for NB (3.3%) [3]. This difference is due to two important extensions; first we have changed the definition of CN-LOH to near-CN-LOH, thus accepting a wider range of abnormalities in order to include the near-triploid tumors. Second, we have extended the dataset by including 62 tumors not included in the first dataset.

The statistical analysis show that near-CN-LOH is more common among NB-cell lines than primary NB tumors (p = 0.002). This could implicate either that CN-LOH is more common among relapsing tumors, from which most cell lines are derived, or that CN-LOH is an effect of cell line immortalization. Near-CN-LOH was also found to be more frequent in the primary tumors than in the healthy controls (p = 0.004), although the healthy controls did present with near-CN-LOH as well. Due to the lack of matched control samples in the material, we were unable to discriminate between IBD and tumor derived CN-LOH in the tumors. However, the difference in frequency seen between these groups indicates that at least some of the near-CN-LOH in the tumors represents true tumor acquired events.

### Possible clinical implications

As can be seen in Figure 2, the primary tumors sometimes display near-CN-LOH in regions of clinical and/or prognostic importance for NB tumors, such as 1 p, 11 q or 17 q. This highlights that even though CN-LOH is not very common in NB, it still adds an extra complexity to tumor genetics, and the total number of aberrations in these regions might be higher than previously thought. Thus by combining CN-LOH data with data about deletions and gains, new regions of interest can be identified and previously characterized regions can be further defined. However, the possible clinical implications of CN-LOH are at the moment difficult to evaluate, ranging from potentially harmless to resulting in tumor suppressor gene silencing, depending on mutational and imprinting status of the remaining allele. Moreover, in contrast to a hemizygous deletion, CN-LOH would not be expected to cause any haploinsufficiency effects, since the total copy number remains normal.

Loss of heterozygosity in combination with gain of the same region (Gain-LOH) is another very intriguing aberration that was found in the material, e.g. in the 17 q region often affected with gain in aggressive NB tumors (Figure 2). This abnormality is especially interesting due to its dual nature, which makes simultaneous promotion of both tumor suppressor genes and oncogenes possible. For some oncogenic gain-of-function mutations, such as the V617F mutation in the tyrosine kinase JAK2 in myeloproliferative disorders, loss of the wild type allele could actually give an advantage to the tumor cell. This mutation targets the auto inhibitory region of JAK2 and results in a constitutively active tyrosine kinase that confers growth factor independency as well as cytokine hypersensitivity. The region surrounding *JAK2 *(9 p) frequently contains CN-LOH or Gain-LOH in these diseases [22-25]. It has also been shown that adding wild type JAK2 to cells carrying the mutated JAK2 allele restores the growth factor dependency, indicating that in this case it is in fact favorable if not necessary for tumor progression to lose the wild type allele [23].

### The subtypes of CN-LOH might represent different mechanisms

The subtypes of CN-LOH were found to differ in their relative frequency between the three sample groups. This probably reflects different modes of acquisition, more or less common in the different groups. The whole chromosome CN-LOH events could be explained by either anaphase lag, causing loss of one allele followed by reduplication, or nondisjunction causing two sister chromatids to end up in the same cell. This could occur either at mitosis as a tumor acquired event or at meiosis as a germ line event [17,26]. Smaller segmental CN-LOH (interstitial and telomeric), on the other hand, probably arise in the tumors either through mitotic recombination between low copy repeats or due to a double-stranded-break repair error followed by reduplication [17].

It is also possible that some of the CN-LOH seen in the tumors might in fact represent IBD and not true tumor acquired events. This mechanism can in theory give rise to any of the different subgroups of CN-LOH. However due to meiotic recombination, the CN-LOH regions would be expected to become smaller with each generation, thus making small, interstitial events more likely than the larger telomeric or whole chromosome events. This could explain at least part of the differences in subtypes seen between the tumors and healthy controls. It is also important to note that although tumor derived CN-LOH is the primary interest here, IBD events might be important for tumor development and act as a first predisposing event towards cancer.

### Genetic differences between cell lines

The array profiles, as well as the STR-genotyping, of the three related cell lines SK-N-SH, SH-EP and SH-SY5Y clearly confirmed that these cell lines are highly related to each other, but some distinct features separating these cell lines from each other was also found (Figure 4). It could be argued that these alterations were all present in a mosaic manner in the parental cell line and perhaps also in the original tumor, and that subcloning later has resulted in more purified cell lines. An alternative hypothesis would be that aberrations found to differ between the cell lines are in fact cell line derived events, and thus a product of cell line evolution. The finding of a gain at 2p16-pter, common to SK-N-SH and SH-SY5Y but not SH-EP give some support to the former theory. However, since each of the three cell lines also contain unique aberrations that differ from the others, it seems unlikely that this theory would account for all of the differences seen. Even though the SK-N-SH line might have lost some of its mosaicism over time, thus becoming a more homogeneous cell line, the fact that extra aberrations such as the deletion of 4q26-28.3 is visible only in SK-N-SH, still indicates that at least some of the aberrations are acquired secondary to the subcloning.

The data also show that both SH-SY5Y and SH-EP contain an extra copy of 1 q, although with different breakpoints and different copy number, thus presumably acquired as two separate events. The parental cell line SK-N-SH however, shows no sign of this aberration in the material, which indicates that this is a later event. According to early karyotypic data for these cell lines [21], SHEP was found to contain an isochromosome 1 q in 35% of the cells, resulting in 4 copies of the 1 q region, while SH-SY5Y presented with a complex duplication of a large 1 q segment, resulting in 3 copies of this region. SK-N-SH however was found to be karyotypically normal for chromosome 1. This corresponds well with the data presented here and explains the observed difference in copy number seen between the cell lines. However, other karyotypic information for these cell lines has also been found in the literature [27-29], resulting in a somewhat complex picture. Although some of these differences can be explained by limitations of the different methods used, for example the lack of resolution in CGH and M-FISH studies or the inability to find balanced translocations using SNP-arrays, there are still some discrepancies between the datasets, suggesting that different clones of these cell lines are used at different laboratories. The fact remains that even relatively stable cell lines change over time and that cell lines keep evolving, thus giving rise to aberrations specific not to the original tumor but rather to the cell culture environment itself. It is important to be aware of the effects that might result from culturing the cells for an extensive amount of passages and to check the cell lines for genetic changes on a regular basis.

Another interesting finding is that karyotypic information in itself sometimes can be misleading. The cell line SK-N-DZ used in this study was recently bought from European Collection of Cell Cultures (ECACC, HPA Culture Collections), where its karyotype is presented as lacking both copies of chromosome 2. However, when this cell line was analyzed with SNP-array it was found that not only does chromosome two exist but a high grade amplification of the *MYCN *gene was also present. The copy number plot for chromosome 2 (except the *MYCN *region) was mainly in the diploid range although somewhat fragmented, indicating that the chromosome might be broken into smaller pieces that are translocated to other regions of the genome and thus hard to detect using karyotyping. In fact the American Type Culture Collection (ATCC) describes the karyotype of this cell line as carrying 5 marker chromosomes of unknown origin, which supports this finding. This highlights that high density SNP-arrays can give information that is complementary to classical karyotype data, and that it is important to compare data from different sources in order to derive full information.

## Conclusions

We have shown that NB tumors harbor several interesting allelic imbalances that would either go unnoticed or be misinterpreted using traditional array CGH methods or other genome-wide techniques. Although the clinical effects of these abnormalities on NB tumor development and progression remains unknown, the findings indicate that the genetics underlying NB might be even more complex than previously known and that SNP arrays are important analysis tools, capable of providing large amounts of information in a single experiment.

We have also shown that these near-CN-LOH events are more frequent in NB cell lines than in NB tumors and that the type of CN-LOH differs between the groups. Furthermore, when analyzing a set of highly related cell lines, several aberrations were found to be unique for each of the subclones which indicate that the cell lines have continued to evolve secondary to the subcloning event. Taken together this highlights that cell lines in many cases differ substantially from the primary tumors they are expected to represent, and that caution should be taken when drawing conclusions from cell line-based studies.

## Authors' contributions

HK and TM initiated the project, performed experimental analyzes, data analyzes, statistical analyzes, co-drafted the manuscript and coordinated the study. HC, JE and RMS aided in the array and molecular experiments. PK and JA provided clinical information. All authors reviewed and approved the final manuscript.

## Supplementary Material

Additional file 1 **Aberrations that differ between the related cell lines**. Table of copy number changes that differs between the related cell lines; SK-N-SH, SH-SY5Y and SH-EP. Gain = 1-3 extra copies, Del = loss of 1 copy (unless stated otherwise in the comments), pter and qter refer to the p- and q-terminal of the chromosome respectively. Click here for file
